# Motives for Cannabis Use and Readiness to Change Among Users of the “Stop-Cannabis” Mobile App: Cluster Analysis

**DOI:** 10.2196/70849

**Published:** 2025-10-03

**Authors:** Milena Wegener, Stéphane Rothen, Elise Dan-Glauser, Tania Lecomte, Stéphane Potvin, Lucien Rochat, Marissa Sjöblom, Germano Vera Cruz, Jean-François Etter, Yasser Khazaal

**Affiliations:** 1 Faculty of Biology and Medicine, University of Lausanne, Lausanne, Switzerland; 2 Department of Mental Health and Psychiatry, Geneva University Hospitals, Geneva, Switzerland; 3 Institute of Psychology, University of Lausanne, Lausanne, Switzerland; 4 Department of Psychology, University of Montreal, Montreal, QC, Canada; 5 Research Center of the Institut Universitaire en Santé Mentale de Montréal, Montreal, QC, Canada; 6 Department of Psychiatry and Addictology, University of Montreal, Montreal, QC, Canada; 7 Department of Psychology, University of Picardie Jules Verne, University of Picardie Jules Verne, Amiens, France; 8 Institute of Global Health, University of Geneva, Geneva, Switzerland; 9 Service de médecine des addictions, University Hospital of Lausanne, Rue du Bugnon 23, Lausanne, 1011, Switzerland, 41 3148400

**Keywords:** cannabis, motives, coping, cannabis problematic use, app, mHealth, mobile phone, mobile health

## Abstract

**Background:**

Cannabis use is widespread and driven by diverse motives, ranging from recreational purposes to coping with psychological distress. Understanding the underlying reasons for cannabis use, their distribution across different subgroups of people who use cannabis, and how they relate to possible behavior change is essential for developing effective prevention and intervention strategies such as smartphone apps designed to support change.

**Objective:**

The primary objective of the study was to determine whether analyzing profiles on the “Stop-cannabis” app (Institute of Global Health, University of Geneva, Switzerland) could reveal subgroups based on motives for cannabis use and readiness to change. A secondary objective was to explore differences among these subgroups in terms of problematic use and other indicators of change readiness.

**Methods:**

This study analyzed data from 2578 individuals using the ”Stop-cannabis app”, a mobile app developed in Switzerland to support those seeking to manage their cannabis use. Participants completed validated questionnaires assessing motives for use (Marijuana Motives Measure [MMM]), readiness to change (Stages of Change Readiness and Treatment Eagerness Scale [SOCRATES]), and risk of problematic use (Alcohol, Smoking, and Substance Involvement Screening Test [ASSIST]). They also self-rated their “readiness for action,” the “importance of change,” and their “confidence in their ability to change.” These assessments were part of the app’s intervention model, with personalized feedback delivered based on participants’ responses; no external incentives were offered. Cluster analysis was conducted to identify subgroups based on MMM and SOCRATES scores.

**Results:**

In total, 3 distinct profiles emerged: the “individually coping users” (ICU), the “social and coping users” (SCU), and the “enhancement-seeking users” (ESU). ICU and SCU scored higher on coping motives compared with ESU, along with greater ambivalence and stronger recognition of problematic use, as measured by SOCRATES. They also scored higher on the ASSIST (indicating greater risk of problematic cannabis use), placed more importance on making behavioral changes, yet reported lower confidence in their ability to enact those changes. By contrast, ESU primarily used cannabis for recreational reasons and had low recognition of problematic use, despite being at moderate risk.

**Conclusions:**

This research highlights that while motives for cannabis use are varied and individually nuanced, distinct subgroups can be identified, each with specific challenges. The findings align with previous research emphasizing the importance of coping motives in behavior change. Tailoring app content to reflect the unique profiles and needs of each subgroup may improve intervention outcomes. For instance, SCU and ICU may benefit from strategies targeting emotion regulation and alternative coping mechanisms, whereas ESU may respond better to brief motivational feedback and harm reduction strategies. Such tailored approaches can enhance the effectiveness of digital tools in promoting meaningful and long-term behavior change.

## Introduction

### Background

With approximately 200 million cannabis users worldwide [1], cannabis is a widely used substance with recognized harmful effects [2], particularly when it is smoked [3]. In Switzerland [4], 3.1% of the population were identified as current users (defined as past-month use), with 20% classified as heavy users engaging in daily or near-daily consumption. Despite this widespread use, few people with cannabis use disorders seek treatment due to various intrinsic and structural barriers [5].

The widespread accessibility of mobile health (mHealth) platforms presents a potential for addressing the treatment gap identified in substance use disorder [6]. The recent trend of developing mental health apps [7] has led to the emergence of a small number of apps focusing on cannabis use [8]. In Switzerland, the “Stop-cannabis” app was developed to be a stand-alone intervention [9]. It provides users with information about cannabis, gives out supportive messages to help them change their cannabis use, helps schedule a cannabis weaning plan, and allows access to an online discussion forum. Encouragingly, preliminary studies indicate users find this app useful [9].

While using the app, the consumers are prompted to complete questionnaires for a better assessment and understanding of their consumption. One of the questionnaires, the Marijuana Motives Measure (MMM) [10], is the most widely used scale to investigate motives of cannabis use. These motives are important because they vary between consumers and are associated with various factors related to the severity of cannabis-related issues [11] as well as different treatment outcomes [12,13].

Readiness to change is a pivotal element in the process of behavioral change, as it mirrors a person’s recognition of their problematic cannabis use and their willingness to modify it. The app evaluates it through the Stages of Change Readiness and Treatment Eagerness Scale (SOCRATES) [14], which is a self-reporting questionnaire. The items the tool explores are articulated around the transtheoretical model of change. This model conceptualizes change as a progression through 5 set stages, extending from the precontemplation of change to its maintenance once change is achieved [15]

The World Health Organization recommends motivational interviewing to support patients through this process of behavioral change. This provenly effective [16] intervention is based on nonjudgmental dialogue [17] that helps individuals find the internal motivation to change their behavior. The “Stop-cannabis” app offers a “Consider Change” self-assessment survey that includes 3 important questions, frequently used as prompts during motivational interviews, asking users to rate their “readiness for action,” the “importance of change,” and their “confidence in [their] ability to change” cannabis use habits.

However, despite the growing availability of digital tools aiming to support cannabis-use–related behavior change, there remains a limited understanding, particularly in naturalistic settings, of who engages with these platforms, what drives their cannabis use, and how ready they are to change. Most existing research focuses either on app efficacy or usage metrics, without sufficiently exploring the heterogeneity of app users in terms of psychological drivers and change readiness. This gap limits the potential for delivering nuanced, individualized interventions through mental health apps. Characterizing users according to their motives for cannabis use and their readiness to change is thus essential. It may open avenues for developing more tailored interventions that could improve outcomes in real-world settings.

### Objective

The primary objective of the study was to determine if analyzing cannabis users’ profiles on the “Stop-cannabis” app could reveal subgroups of consumers based on their motives for cannabis use and their readiness to change said use. The second objective was then to study the characteristics of these subgroups, such as problematic cannabis use risk, while exploring their potential for change through factors such as “readiness for action,” perceived “importance of change,” and “confidence in one’s ability to change.”

## Methods

### Recruitment

Participants in the study were individuals who chose to download the app spontaneously. The app can be obtained at no cost from the Google Play Store (Android) and the App Store (iOS), and it does not include any commercial advertisement. Once downloaded, users were invited to anonymously provide their age and gender, after which they were prompted to use the Alcohol, Smoking, and Substance Involvement Screening Test (ASSIST) tool to assess their cannabis use. This enabled them to get personalized feedback and to customize the app. Users could also get feedback through an automated motivational interview feature, which used the MMM and SOCRATES surveys, as well as the “Consider Change” self-rating survey.

In this study, we performed a secondary analysis of data that had been previously used in a published study about the motives of cannabis use in cannabis consumers [18].

### Participants

Between September 20, 2010, and January 13, 2020, 4077 user profiles for individuals from French-speaking countries (Switzerland, France, Canada, Belgium, and Luxembourg) were created on the app, and these individuals completed questionnaires provided in the app. Upon analysis, 95 duplicate user profiles and 450 underage (<18 y old) user profiles were excluded, leaving 3532 individuals’ user profiles as the study sample. Of these, 2578 (73%) fully completed the ASSIST, SOCRATES, MMM, and “Consider Change” surveys and were selected as the analyzed sample (N). We did not consider user age and gender in this study. Users completed these questionnaires as part of their natural engagement with the app’s functionalities. They were not offered any incentives and could complete the questionnaires at any time after downloading the app, at their own discretion and pace.

### Measures-Questionnaires

#### MMM

The MMM is a 25-item self-report questionnaire that is divided into 5 subscales. It was developed in 1998 [10] and subsequently validated in different samples of young adult marijuana users [19,20]. Each of the 5 subscales explores a different motive for cannabis use: “coping,” “enhancement,” “social,” “conformity,” and “expansion.” Coping refers to using cannabis to relieve negative affective states (eg, “to forget my problems or my worries”). Enhancement means the substance is used to provide positive sensations or emotions (eg, “because it’s fun,” “I like the feeling”). Social motive indicates cannabis use together with peers to enhance a positive situation (eg, “because it improves parties or social gatherings”). Conformity alludes to its use to bond and avoid social rejection (eg, “to fit in with the group”). Expansion refers to the use of cannabis as a form of mind expansion (eg, “to expand my awareness,” “to understand things differently”). Items are rated on a 5-point scale ranging from 1 (almost never or never) to 5 (almost always or always), the higher scores indicating a greater agreement with the motive.

#### SOCRATES

This tool was initially developed based on a cohort of adults with alcohol use disorders [14] but was adapted to other drugs in a later version (designated 8D). It assesses both preparedness for change in drug use habits and motivation to partake in treatment. In its current form, it consists of 19 self-reporting items, rated from 1 (strongly disagree) to 5 (strongly agree). The items explore 3 dimensions: “recognition,” “ambivalence,” and “taking steps.” “Recognition” refers to the cognitive awareness of the problems caused by the substance use (eg, “I have serious problems with cannabis.”). “Ambivalence” represents an individual’s level of certainty about having a substance use problem (eg, “There are times when I wonder if I use too much cannabis.”). “Taking steps” evaluates already implemented behavioral changes (eg, “I am actively doing things now to cut down or stop cannabis use.”). The resulting scores are expressed in deciles, from 10 (very low) to 90 (very high).

#### ASSIST

This tool [21-23] was developed by the World Health Organization to screen for problematic substance use. It includes subscales for different substances, with the marijuana subscale examining use in the last 3 months. The severity of problematic cannabis use is assessed over 7 questions. Frequency of use, presence of craving, impact on health, and negative social consequences (eg, legal and financial problems) are rated on a 5-point Likert scale ranging from 0 (“never in the past 3 months”) to 4 (“daily or almost daily”). Failure to perform daily responsibilities, concern expressed by relatives, and failure to control, cut down, or stop cannabis use are rated on a 3-point Likert scale (with 0=“no, never,” 1=“yes in the past 3 months,” and 2=“yes but not in the past 3 months”). The compounded score ranges from 0 to 39, with a score above 27 indicating a high risk of dependence and of adverse health or social outcomes from the substance use.

#### “Consider Change” Survey

A total of 3 questions that are frequently asked in motivational interviews were combined to form a self-assessment tool. “Readiness for action,” the “importance of change,” and “confidence in one’s ability to change” were rated on a visual scale and then converted into scores from 0 to 100.

### Ethical Considerations

All participants provided informed online consent before data collection, explicitly agreeing that their anonymized data could be stored and used for research purposes. Only data from consenting participants were included in the study. Completion of the surveys was an integral part of the app intervention system and provided users with automated personalized feedback on their cannabis use. In addition, the data served to monitor app quality, as required by the funding source (Canton of Geneva).

All assessments were collected and reported anonymously. According to the Swiss Human Research Act (Chapter 1, Section 1, Article 2, Scope: 2c) [24], studies involving the collection of fully anonymized health-related personal data are exempt from ethical approval requirements. Therefore, this survey did not require submission to an ethics committee. The study was conducted in accordance with the principles of the Declaration of Helsinki.

### Statistical Analysis

We conducted a cluster analysis on the 5 MMM subscales and the 3 SOCRATES subscales. Initially, we assessed the suitability of the data for clustering analysis using the Hopkins statistic [25]. Cluster analysis, in contrast to traditional linear models like regression, highlights individual differences [26] and enables the evaluation of complex, nonlinear interactions, thus offering enhanced ecological validity. Indeed, linear models might overlook significant relationships that are relevant for some individuals but not for all [27].

Traditionally, cluster analysis has often been conducted using a single method, typically the k-means method. However, with the diverse range of clustering methods available, it is essential to explore multiple approaches as no single method may be universally optimal [26]. Hence, five clustering methods were used: (1) k-means (with 50 starting points to mitigate the risk of local minima), (2) partitioning around medoids, (3) single linkage hierarchical clustering, (4) complete linkage hierarchical clustering, and (5) average linkage hierarchical clustering. The range of clusters considered for each method was set from 2 to 10, resulting in a total of 45 clusterings. Subsequently, we chose the optimal solution.

To determine the optimal number of clusters and clustering method for the dataset, we used a composite index based on 4 key characteristics of clustering [28]. These characteristics include (1) average distance within clusters, assessing the similarity of observations within each cluster; (2) separation index, ensuring distinctiveness between clusters; (3) Pearson-Gamma, measuring clustering quality in terms of dissimilarity structure approximation [29]; and (4) entropy of cluster membership distribution, aiming for clusters of equivalent size [30]. We used the R function clusterbenchstats to generate standardized validity index values for comparison across indexes.

Statistical analysis was done on R-Studio 2023.12.1.402 [31] using R 4.3.3 [32]. We computed the composite index with the clusterbenchstats function from the fpc library [33]. All data and R scripts are available on request to the corresponding author.

## Results

### Cluster Model

Hopkins statistic values above 0.75 indicate a strong clustering tendency [34]. In this sample, the Hopkins statistic was 0.99, which confirms that cluster analysis is suitable in this sample. Based on the composite index, the optimal clustering solution was found for the k-means methods with 3 or 6 clusters. We selected the 3-cluster solution because the 6-cluster solution was not clinically meaningful, with an absence of significant difference between the clusters and a small cluster composed of only 130 individuals (Multimedia Appendix 1). In the 3-cluster solution, cluster 1 is composed of 43.87% (1131/2578) of user profiles, cluster 2 of 26.1% (674/2578), and cluster 3 of 29.9% (773/2578) profiles (Multimedia Appendix 2).

### Clusters’ Characteristics

#### Description of the 3 Clusters Regarding MMM 5 Factors’ Scores

**Figure 1.**
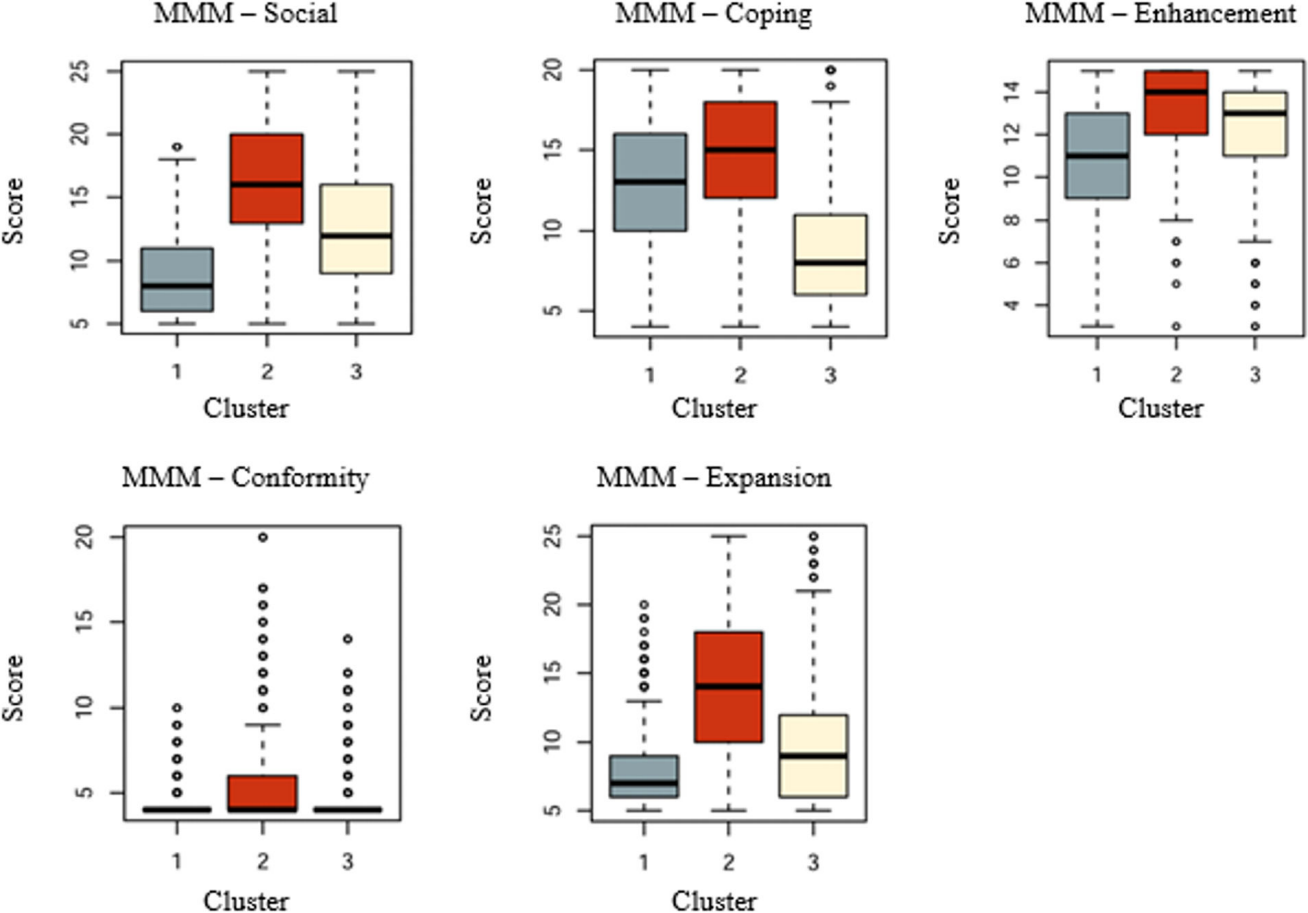
Representation of MMM 5 factors’ scores for the 3 clusters. MMM: Marijuana Motives Measure.

Cluster 1 (1131/2578, 43.87% of the sample) is characterized by low social (median 8/25, IQR 6-11/25) and expansion (median 7/25, IQR 6-9/25) motive scores compared with clusters 2 and 3, with a high score on coping (median 13/20, IQR 10-16/20) compared with cluster 3 (Figure 1, Table 1). Enhancement, while high (median 11/15, IQR 9-13/15), is slightly lower than the 2 following clusters, and conformity is low (median 4/20, IQR 4-4/20).

The individuals within cluster 1 can be described as “individually coping users” (ICU). They resort to cannabis consumption principally for the self-regulation of negative emotions, with a high score in coping, but a low score for social, conformity, and expansion motives of use.

Cluster 2 (674/2578, 26.1%) is also characterized by a high score on coping (median 15/20, IQR 12-18/20) and enhancement (median 14/15, IQR 12-15/15), but the users differ from the ones of cluster 1 as they present higher scores on social (median 16/25, IQR 13-20/25) and expansion motives (median 14/25, IQR 10-18/25). While minimal, the conformity motive score has a higher tendency (median 4/20, IQR 4-6/20) than the other 2 groups.

Cluster 2 is more heterogeneous and comprises individuals who can be described as “social and coping users” (SCU). Their cannabis use predominantly revolves around social aspects, enhancing social interactions (highest score in social motive) and reinforcing social cohesion (relatively high conformity), while in parallel serving as a pivotal coping mechanism (highest coping scores). Their motives for cannabis use are complex as they also score the highest in enhancement and expansion.

Cluster 3 (773/2578, 29.9%) includes users with low coping scores (median 8/20, IQR 6-11/20) and high enhancement scores (median 13/15, IQR 11-14/15). The social motive scores are moderate (median 12/25, IQR 9-16/25), higher than cluster 1 but lower than cluster 2. Expansion (median 9/25, IQR 6-12/25) and conformity (median 4/20, IQR 4-4/20) scores are low.

The consumers in cluster 3 can be described as “enhancement-seeking users” (ESU), their most salient motive being recreational (enhancement of positive activities). Coping is only a minor reason for use, with social and expansion components also being of secondary interest and conformity not being a concern.

**Table 1.** Marijuana Motives Measure (MMM) 5 factors’ scores for the 3 clusters and comparison.

MMM factor	Cluster 1 (ICU^a^; n=1131), median (IQR)	Cluster 2 (SCU^b^; n=674), median (IQR)	Cluster 3 (ESU^c^; n=773), median (IQR)	*P* value^d^
Social	8 (6-11)	16 (13‐20)	12 (9‐16)	<.001^efg^
Coping	13 (10‐16)	15 (12‐18)	8 (6-11)	<.001^efg^
Enhancement	11 (9‐13)	14 (12‐15)	13 (11‐14)	<.001^efg^
Conformity	4 (4-4)	4 (4-6)	4 (4-4)	<.001;^eg^ .15^f^
Expansion	7 (6-9)	14 (10‐18)	9 (6-12)	<.001^efg^

a ICU: individually coping users.

b SCU: social and coping users.

c ESU: enhancement-seeking users.

d Kruskal-Wallis rank sum test.

e Post hoc test comparing cluster 1 and cluster 2, Bonferroni adjusted *P* value <.05.

f Post hoc test comparing cluster 1 and cluster 3, Bonferroni adjusted *P* value <.05.

g Post hoc test comparing cluster 2 and cluster 3, Bonferroni adjusted *P* value <.05.

#### Description of the 3 Clusters Regarding SOCRATES Scores

**Figure 2.**
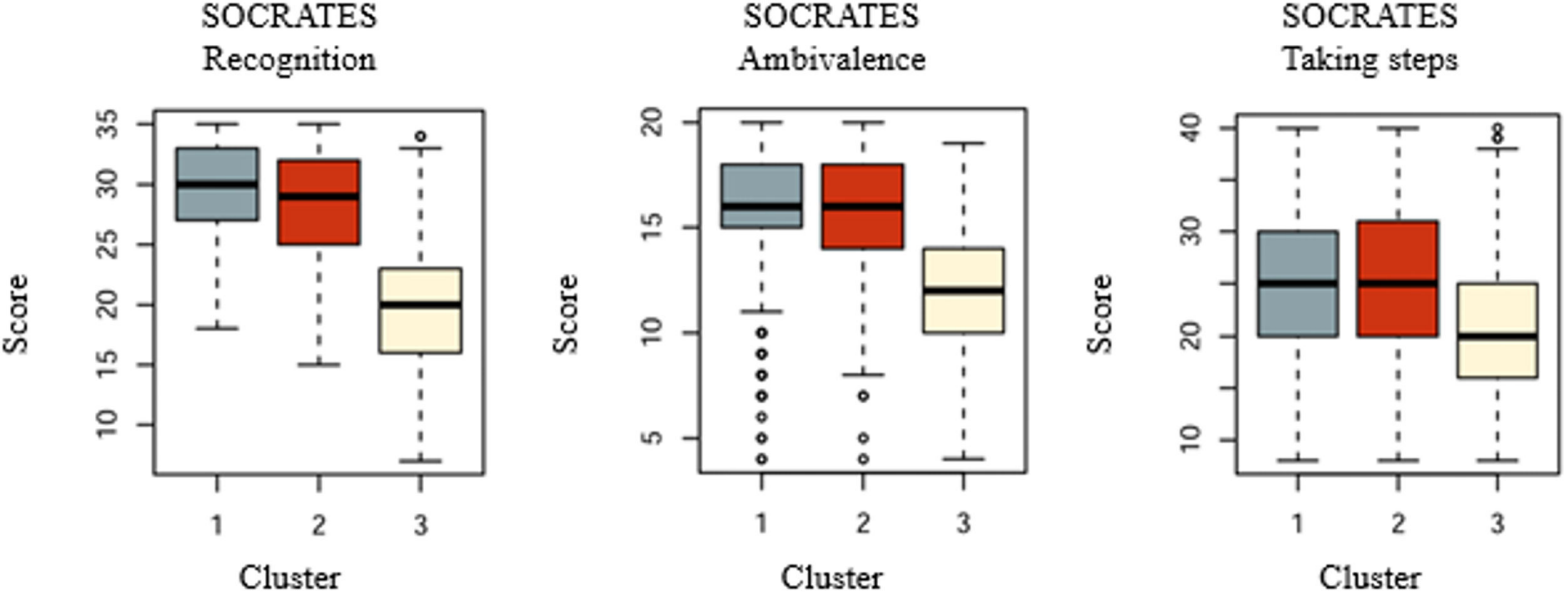
Representation of SOCRATES subscales between the 3 clusters. SOCRATES: Stage of Change Readiness and Treatment Eagerness Scale.

Cluster 1 (ICU) is characterized by high recognition score (median 30/35, IQR 27-33/35) and ambivalence (median 16/20, IQR 15-18/20) scores, compared with cluster 3 (Figure 2, Table 2). “Taking steps” score (median 25/40, IQR 20-30/40) was moderate.

Cluster 2 (SCU) follows a similar pattern, with high recognition (median 29/35, IQR 25-32/35) and ambivalence (median 16/20, IQR 14-18/20) scores, while “taking steps” score (median 25/40, IQR 20-31/40) was moderate.

Cluster 3 (ESU) differs in presenting with the lowest recognition (median 20/35, IQR 16-23/35) and ambivalence (median 12/20, IQR 10-14/20) scores, and slightly lower “taking steps” scores (median 20/40, IQR 16-25/40) compared with the other 2 groups.

**Table 2.** Stage of Change Readiness and Treatment Eagerness Scale (SOCRATES) subscale scores for the 3 clusters and comparison.

SOCRATES subscale	Cluster 1 (ICU^a^; n=1131), median (IQR)	Cluster 2 (SCU^b^; n=674), median (IQR)	Cluster 3 (ESU^c^; n=773), median (IQR)	*P* value^d^
Recognition	30 (27‐33)	29 (25‐32)	20 (16‐23)	<.001^ef g^
Ambivalence	16 (15‐18)	16 (14‐18)	12 (10‐14)	<.001^fg^; >.99^e^
Taking steps	25 (20‐30)	25 (20‐31)	20 (16‐25)	<.001^fg^; .50^e^

a ICU: individually coping users.

b SCU: social and coping users.

c ESU: enhancement-seeking users.

d Kruskal-Wallis rank sum test.

e Post hoc test comparing Cluster 1 and Cluster 2, Bonferroni adjusted *P* value <.05.

f Post hoc test comparing Cluster 1 and Cluster 3, Bonferroni adjusted *P* value <.05.

g Post hoc test comparing Cluster 2 and Cluster 3, Bonferroni adjusted *P* value <.05.

#### Description of the Other Clinical Measures in the 3 Clusters

Significant effects of cluster belonging were obtained on all other clinical measures (ASSIST and “Consider Change” survey), between clusters 1 and 2 compared with cluster 3. Between clusters 1 and 2, only “importance of change” was significantly different (Table 3).

Most participants (Multimedia Appendix 3) scored into the moderate (score between 4 and 26) and high-risk (score more than 27) cannabis use groups, according to ASSIST scores [14]. The median ASSIST score for the whole study sample was 27 (IQR 21‐32), which is the cutoff between moderate and high-risk groups.

**Table 3.** Association between clusters and other clinical measures.

Clinical measure	Cluster 1 (ICU^a^; n=1131), median (IQR)	Cluster 2 (SCU^b^; n=674), median (IQR)	Cluster 3 (ESU^c^; n=773), median (IQR)	*P* value^d^
ASSIST^e^	30 (25‐33)	30 (25‐34)	21 (15‐26)	<.001^gh^
“Consider Change”				
Importance of change	99 (80‐99)	90 (74‐99)	66 (46‐80)	<.001^fg h^
Readiness for action	80 (62‐99)	80 (64‐99)	68 (40‐81)	<.001^gh^
Confidence in one’s ability to change	43 (24‐70)	41 (22‐68)	52 (30‐80)	<.001^gh^

a ICU: individually coping users.

b SCU: social and coping users.

c ESU: enhancement-seeking users.

d Kruskal-Wallis rank sum test.

e ASSIST: Alcohol, Smoking and Substance Involvement Screening Test.

f Post hoc test comparing cluster 1 and cluster 3, Bonferroni adjusted *P* value <.05.

g Post hoc test comparing cluster 1 and cluster 2, Bonferroni adjusted *P* value <.05.

h Post hoc test comparing cluster 2 and cluster 3, Bonferroni adjusted *P* value <.05.

ICU and SCU shared identical median ASSIST scores (median 30, IQR 25‐34), which were significantly higher than those of ESU (median 21, IQR 15‐26).

Cluster 1 (ICU) scored the highest when rating the “importance of change,” with a significantly higher score than cluster 2 (SCU). Both groups also scored significantly higher than cluster 3 (ESU).

Regarding “readiness for action,” clusters 1 (ICU) and 2 (SCU) scored similarly but significantly higher than cluster 3 (ESU).

Cluster 3 (ESU) achieved the highest scores in “confidence in one’s ability to change,” significantly surpassing clusters 1 (ICU) and 2 (SCU).

## Discussion

### Results Analysis

The aim of this study was to identify profiles of cannabis consumers based on their motives for use and their stage of change readiness and treatment eagerness. Both statistically significant and clinically relevant consumer groups were identified: ICU, SCU, and ESU.

Both ICU and SCU clusters disclosed largely using cannabis as a coping mechanism, which in cannabis users has been linked with poorer mental health, significant psychopathological symptoms, and increased psychological stress compared with noncannabis users [35]. Both groups also reported high ASSIST scores, indicating a high risk of problematic cannabis use, but with relatively high levels of problem recognition. This recognition, combined with high willingness to act scores and great importance given to change, offers opportunities for targeted health interventions.

Within this perspective, understanding the underlying coping motives may play a critical role. For instance, in this study, coping motives are positively associated with problematic use—as measured by the ASSIST—and this overlap was reflected in our clustering results. The assessment of motives provides, however, conceptually and clinically distinct information. The ASSIST captures the severity and consequences of substance use, whereas coping motives reflect why individuals engage in cannabis use in the first place. This distinction is critical because motives can serve as predictive and explanatory variables, informing not just current use severity but also potential relapse risk, underlying psychological vulnerabilities (eg, emotion regulation difficulties), and targets for intervention. In particular, coping motives have consistently emerged as one of the strongest and most reliable psychological predictors of both cannabis use frequency and cannabis-related problems [11,18]. Importantly, 2 individuals with similar ASSIST scores might differ in their underlying motives—one using cannabis primarily to cope with distress, and another for social or enhancement reasons—which may call for different intervention strategies. Thus, including motivational profiles allows for a more personalized and mechanism-based understanding of users, which is particularly relevant in the context of digital health tools aiming to offer tailored, scalable intervention.

As it stands, “taking steps” scores, according to the SOCRATES scores, remained moderate in ICU and SCU clusters, probably hampered by high ambivalence and low self-confidence to change. These last 2 factors may reflect pre-existing psychopathological symptoms or psychosocial distress, with cannabis being used to cope with and alleviate these symptoms [35-40]. This dimension should probably be specifically addressed for better therapeutic efficacy. In that respect, research has shown that reducing coping as a motive for cannabis use is a consistently positive predictive factor for a decrease in cannabis-related problems over the next months during treatment [13].

The ESU cluster reported the lowest ASSIST scores, which still translate into a moderate risk of problematic cannabis use. While low coping scores hint at a lower prevalence or burden of psychological comorbidity, individuals are still at risk for the negative physical consequences of smoking cannabis [3]. In other studies, enhancement motives remained predictive of psychosocial problems associated with cannabis use [41]. While enhancement was a highly endorsed motive across all clusters, it still holds discriminative value, as the clusters differed significantly on this dimension—cluster 2 scored highest, cluster 1 lowest, and cluster 3 intermediate. Notably, in cluster 3, enhancement was the most prominent motive relative to other MMM dimensions, justifying its label as the “enhancement-seeking” cluster.

Recognition of the problem was the lowest in the ESU cluster among the 3 groups, and in regard to this, low ambivalence scores should be interpreted as reflecting low awareness of the problem. These results could suggest that such individuals might be more likely to return to the pre-contemplation stage of change despite having downloaded an app aimed at reducing or stopping their use of cannabis. In this case, motivational dialogue might help increase problem recognition and the overcoming of resistance to change. ESU individuals have higher self-confidence in changing their cannabis use, which may be related to lower reliance on cannabis as a coping mechanism for psychological distress.

The SCU cluster was the most intricate, with several salient motives for cannabis use, rendering prognosis analyses and planning treatment interventions more complex. In another study, young adults with social motives for use reported similar levels of distress to nonusers [35]. Notwithstanding these results, evidence suggests that multiplicity of motives for use correlates with increased consumption and a higher likelihood of experiencing adverse consequences of cannabis [42]. SCU also exhibited high rates of use for expansion motives. A decrease in expansion as a reason for cannabis use following treatment is surprisingly associated with poorer treatment outcomes [12].

The distinct features of the 3 main clusters clearly illustrate the multifaceted nature of cannabis use, highlighting diverse motives for use and hence the need for tailored targets of intervention. Targeted therapeutic interventions rely on such multidimensional phenotypic assessments [43] to enhance their efficiency. Motives of use offer a good treatment target, as different motives are associated with either favorable or unfavorable treatment outcomes [12]. As an example, cognitive behavioral therapy, which is sometimes dispensed through computer-based training, could be enhanced by the adjunction of specific modules designed to change motives of cannabis use [44].

Treatment strategies may be effectively tailored according to user profiles identified through clustering. For example, individuals in the ICU cluster appear to use cannabis primarily to cope with psychological distress. They show high recognition of the problem and a strong willingness to change, but report low self-confidence and only moderate levels of action-taking. For this group, tailored interventions could focus on emotion regulation, stress management, and the development of alternative coping strategies. In addition, motivational enhancement techniques may be used to strengthen self-efficacy and support-maintained behavior change.

Individuals in the ESU cluster tend to use cannabis for excitement, exhibit low recognition of the problem, low ambivalence, but express high confidence in their ability to change. In this case, brief interventions—such as personalized assessments and normative feedback—may be appropriate. These should help participants explore the discrepancy between their current behavior and personal goals. Harm reduction strategies (eg, promoting safer cannabis use) should also be offered to support goal-congruent outcomes.

Members of the SCU cluster demonstrate multiple motivations for use (coping, social, and expansion), combined with a high willingness to change but low self-confidence. A multicomponent treatment plan should be considered, including social skills training (to address socially motivated use) and emotion regulation techniques. Motivational interviewing, harm reduction strategies, and interventions aimed at enhancing self-confidence are also recommended. Peer support groups may further provide meaningful sources of social validation and connection.

These cluster-specific considerations could inform the development of future app-based interventions, enabling more precise and multimodal tailoring of treatment. Such proposals merit empirical evaluation in future studies.

### Limitations

The app collected no data on the overall chronology of an individual’s cannabis use, both regarding its overall duration (eg, to distinguish long-term chronic users) and possible time-dependent fluctuations in it. Consumer age was also not considered in the study design. Motives for cannabis use might therefore vary depending on consumer age (eg, phases of life) and their life-long history of use (new users vs chronic users), potentially providing an alternative explanation for the significant differences between the clusters. Fundamentally, this does not influence targets for intervention but might alter their modalities. Gender was not included as a variable in this study design and should be investigated further, as women tend to have higher coping scores and lower social motives scores than men [18].

A further limitation of this study is the absence of precise information regarding when, during their engagement with the app, users completed the questionnaires. As survey completion was anonymous, voluntary, and untied to specific stages of app use, we cannot determine whether responses were given at the outset, during active use of behavior change tools, or following exposure to features such as the within-app social network or psychoeducation content. These interactions may have already influenced the answers of the participants. Nevertheless, the findings revealed meaningful differences between user profiles, underscoring the heterogeneity of app users and suggesting varied needs that could be addressed through more tailored interventions.

This cross-sectional study yielded promising results, but further study, including studies with longitudinal designs, is warranted. In addition, due to the design of the study, participants were spontaneous users of a mobile app, and it is therefore unclear if the results can be extrapolated to the general population.

In addition, the choice of a more robust 3-cluster model rather than a model with a higher number of clusters can conceal other subtypes of consumers. The SCU cluster might be a composite subgroup based on its intrinsic complexity and diversity of motives.

Finally, the very salient coping motive should also be further explored, with more detailed exploration of subgroups of anxiety-coping, depression-coping, and boredom-coping [45]. Numerous studies have shown both the clinical significance and the heterogeneity of coping as a motive [38-40]. Cannabis use as a therapeutic intervention for sleep disorders [40,46] or medical conditions (eg, chronic pain) was also not explored as a possible motive. Be it medically prescribed or self-administered, cannabis use in this setting has also been linked to problematic use [47]. However, given the increasing global use of cannabis for medicinal purposes, including in some of the countries from where the participants come from (ie, Canada and Switzerland), it is possible that coping-related items capture not only emotional coping but also elements of self-medication for physical or psychological symptoms. In the absence of explicit questions related to medical cannabis use, this study cannot disentangle therapeutic motives from other coping motives.

### Conclusions

In this study, we identified 3 distinct profiles of individuals engaging with a cannabis behavior change app: the ICU, the SCU, and the ESU.

ICU and SCU demonstrated higher coping motive scores compared with ESU, along with greater ambivalence and stronger recognition of their problematic use, as measured by the SOCRATES questionnaires. In addition, they scored higher on the ASSIST, placed greater importance on making behavioral changes, yet reported lower confidence specifically in their ability to enact those changes. These findings align with existing research emphasizing the critical role of coping motives in behavior change and highlight the need to tailor the content and design of digital interventions to better address the unique needs and characteristics of these distinct user subgroups. By considering these clusters, digital tools can become more effective in supporting behavior change and achieving long-term success for diverse populations seeking help.

The heterogeneity of cannabis use is a challenge for researchers and practitioners alike, and this study showed that a cluster approach is a useful method for discovering different profiles of users. mHealth approaches are proving useful for research as they facilitate the collection of standardized data in high numbers.

## Supplementary material

10.2196/70849 Multimedia Appendix 1 Six-cluster solution.

10.2196/70849 Multimedia Appendix 2 Three-cluster model repartition.

10.2196/70849 Multimedia Appendix 3 ASSIST scores and corresponding risk levels in the sample. ASSIST: Alcohol, Smoking, and Substance Involvement Screening Test.

## Abbreviations

ASSIST: Alcohol, Smoking, and Substance Involvement Screening Test
ESU: enhancement-seeking users
ICU: individually coping users
mHealth: mobile health
MMM: Marijuana Motives Measure
SCU: social and coping users
SOCRATES: Stages of Change Readiness and Treatment Eagerness Scale
