# Supplementary figures and images for: Motives for Cannabis Use and Readiness to Change Among Users of the “Stop-Cannabis” Mobile App: Cluster Analysis
Source: JMIR Form Res. 2025 Oct 3;9:e70849. doi: 10.2196/70849 (PMC12494107; doi:10.2196/70849)

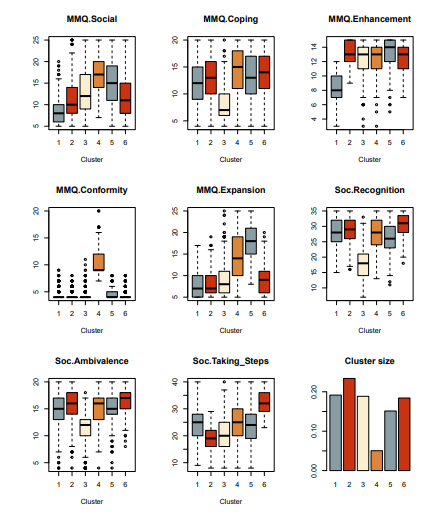

Supplement: Multimedia Appendix 1 [file formative-v9-e70849-s001.png]

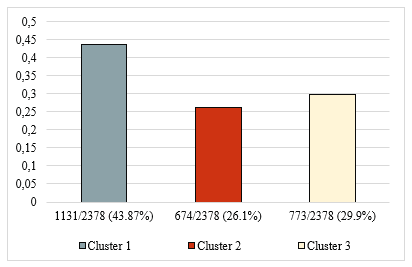

Supplement: Multimedia Appendix 2 [file formative-v9-e70849-s002.png]

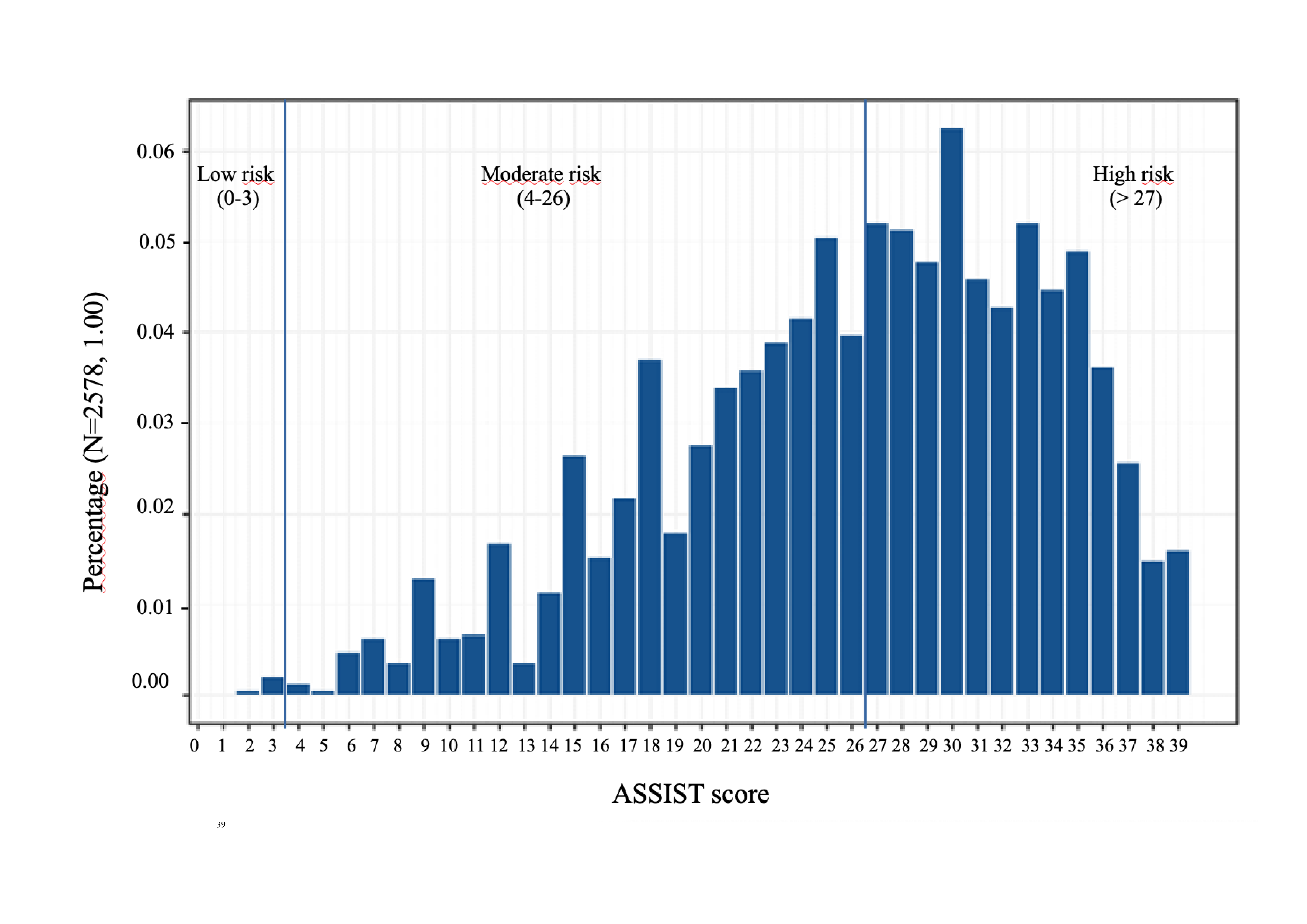

Supplement: Multimedia Appendix 3 [file formative-v9-e70849-s003.png]
